# Declines in exercise performance are prevented 24 hours after post-exercise ischemic conditioning in amateur cyclists

**DOI:** 10.1371/journal.pone.0207053

**Published:** 2018-11-09

**Authors:** Rhaí André Arriel, Hiago Leandro Rodrigues de Souza, Gustavo Ribeiro da Mota, Moacir Marocolo

**Affiliations:** 1 Department of Physiology, Federal University of Juiz de Fora, Juiz de Fora, Minas Gerais, Brazil; 2 Human Performance and Sport Research Group, Department of Sport Sciences, Federal University of Triângulo Mineiro, Uberaba, Minas Gerais, Brazil; Sao Paulo State University - UNESP, BRAZIL

## Abstract

Brief moments of blood flow occlusion followed by reperfusion may promote enhancements in exercise performance. Thus, this study assessed the 24-h effect of post-exercise ischemic conditioning (PEIC) on exercise performance and physiological variables in trained cyclists. In a randomized, single-blind study, 28 trained cyclists (27.1 ± 1.4 years) performed a maximal incremental cycling test (MICT). The outcome measures were creatine kinase (CK), muscle soreness and perceived recovery status, heart rate, perceived exertion and power output. Immediately after the MICT, the cyclists performed 1 of the following 4 interventions: 2 sessions of 5-min occlusion/5-min reperfusion (PEIC or SHAM, 2 x 5) or 5 sessions of 2-min occlusion/2-min reperfusion (PEIC or SHAM, 5 x 2). The PEIC (50 mm Hg above the systolic blood pressure) or SHAM (20 mm Hg) treatment was applied unilaterally on alternating thighs. At 24 h after the interventions, a second MICT was performed. In all the groups, the CK levels were increased compared with the baseline (p < 0.05) after the 24-h MICT. The PEIC groups (2 x 5 and 5 x 2) felt more tired at 24 h post intervention (p < 0.05). However, both PEIC groups maintained their performance (2 x 5: p = 0.819; 5 x 2: p = 0.790), while the SHAM groups exhibited decreased performance at 24 h post intervention compared to baseline (2 x 5: p = 0.015; 5 x 2: p = 0.045). A decrease in the maximal heart rate (HR) was found only in the SHAM 2 x 5 group (p = 0.015). There were no other significant differences in the heart rate, power output or perceived exertion after 24 h compared with the baseline values for any of the interventions (p > 0.05). In conclusion, PEIC led to maintained exercise performance 24 h post intervention in trained cyclists.

## Introduction

Road cycling and cross-country mountain biking are modalities characterized by several competitions each season. Each competition commonly consists of consecutive races (e.g., two or eight days) lasting 115–240 min each day [1,2]. During these high-intensity competitions, the athletes deal with short recovery periods between trials (e.g., 24 h or less), which could decrease subsequent performance (i.e., hours or days later) [3].

With the goal of achieving better results, different post-exercise recovery strategies have been applied to attenuate fatigue, performance decrements, and muscle soreness [4] and to accelerate recovery for training sessions or subsequent competitions [5]. Among these strategies, short periods of limb ischemia followed by reperfusion (IR), applied before exercise, have shown beneficial effects in maximizing sports performance [6]. However, few studies have investigated the effectiveness of IR, applied post-exercise (PEIC) on performance [7–9]. Beaven et al. [7] found improvements in performing countermovement and squat jumps after 24 h of recovery, applying two 3-min cycles of PEIC. However, Northey et al. [8] used this protocol (i.e., 2 cycles of 3 min each) and found no improvements in countermovement and squat jumps 24 h after a fatiguing resistance exercise bout. Nevertheless, Page et al. [9] applied three 5-min cycles of PEIC after exercise-induced muscle damage and thereby significantly attenuated a decrease in the maximal isometric voluntary contraction 24, 48 and 72 h after the PEIC intervention.

Originally, the IR interventions, demonstrated cardiac and skeletal injury protection after prolonged ischemic insult [10], which is associated with an increase in tissue biomarkers (e.g., creatine kinase) [10] and oxidative damage [11]. These markers are similar to those found after cycling exercise [12] and professional cycling races [3]. Thus, we speculated that PEIC intervention, through elevated levels of nitric oxide (NO), could attenuate tissue and oxidative injury after metabolic and muscle stresses caused by cycling exercise. NO directly reacts with reactive oxygen species (ROS) or inhibits mitochondrial ROS generation [11]. NO also limits tissue injury [11,13] and reduces blood creatine kinase (CK) levels [10]. Page et al. [9] applied PEIC intervention after a bout of exercise and found decreased markers of muscle damage (e.g., CK) in recreationally active males. Moreover, clinical research suggests that the effects of IR occur in two phases [14]: the early phase, which starts immediately after reperfusion and lasts between 3 and 4 h; and the late phase, which begins between 12 and 24 h after intervention. It is believed that the IR intervention effect is prolonged (i.e., the later phase) by recruiting several complex signal transduction pathways [15].

Since the effects of PEIC are still unclear, this study assessed the PEIC effect on exercise performance and physiological variables 24 h post intervention in trained cyclists. We hypothesized that PEIC would improve performance 24 h after engaging in a cycling exercise until exhaustion.

## Materials and methods

### Participants

This study was approved by the Federal University of Juiz de Fora Ethics Committee (Number 2.250.458) for human experiments and was performed in accordance with the Declaration of Helsinki. Based on a prior sample-size calculation (effect size: 0.8; test power: 0.8), it was estimated that a total of 24 subjects were required. However, we included one more subject in each group, considering the possible withdrawal of a volunteer. Therefore, twenty-eight male cyclists voluntarily participated in this research. All the cyclists were healthy and trained; for inclusion, they needed to be able to reach at least 250 W or more in the incremental test [16]. Table 1 shows the participants’ characteristics. The exclusion criteria were: i) any cardiovascular or metabolic disease; ii) caffeine supplement usage; iii) use of exogenous drugs, anabolic-androgenic steroids or any potential substance that could promote improvement in exercise performance; iv) smoking history; and v) musculoskeletal, bone or joint injury that could limit the proposed exercise performance. This information was identified by the self-reporting of the participants via a questionnaire. Additionally, all the subjects signed an informed consent form.

**Table 1:** Demographic and anthropometric characteristics of volunteers.

Characteristics	PEIC 2 x 5 (n = 7)	SHAM 2 x 5 (n = 7)	PEIC 5 x 2 (n = 7)	SHAM 5 x 2 (n = 7)	P_values_
Age (years)	27.6 ± 4	27.8 ± 4.2	25 ± 4.5	28.3 ± 2.3	0.456
Height (cm)	177 ± 4.7	175.6 ± 2.8	176 ± 5.6	174.7 ± 1.8	0.913
Body mass (kg)	80.3 ± 10.7	77.8 ± 7.5	76 ± 8.7	74.6 ± 6.2	0.560
Body fat (%)	16.2 ± 5.3	16.5 ± 3.3	14.2 ± 4.1	14.2 ± 2.8	0.371
PO_peak_ (W)	315 ± 32.2	315.2 ± 44.5	327 ± 55.6	302.3 ± 52.0	0.816
PO_peak_ (W.kg^-1^)	4.0 ± 0.8	4.0 ± 0.3	4.3 ± 0.5	4.1 ± 0.7	0.777
***Training History***					
Experience (years)	3.2 ± 1.7	3.1 ± 1.5	3.3 ± 1.7	3.0 ± 1.7	0.924
Hours per week	5.1 ± 2.3	4.9 ± 1.1	4.7 ± 1.7	4.2 ± 2.0	0.890

PEIC = Post-exercise ischemic conditioning; SHAM = cuff administration with low pressure; PO = power output.

### Experimental design

**Fig 1:**
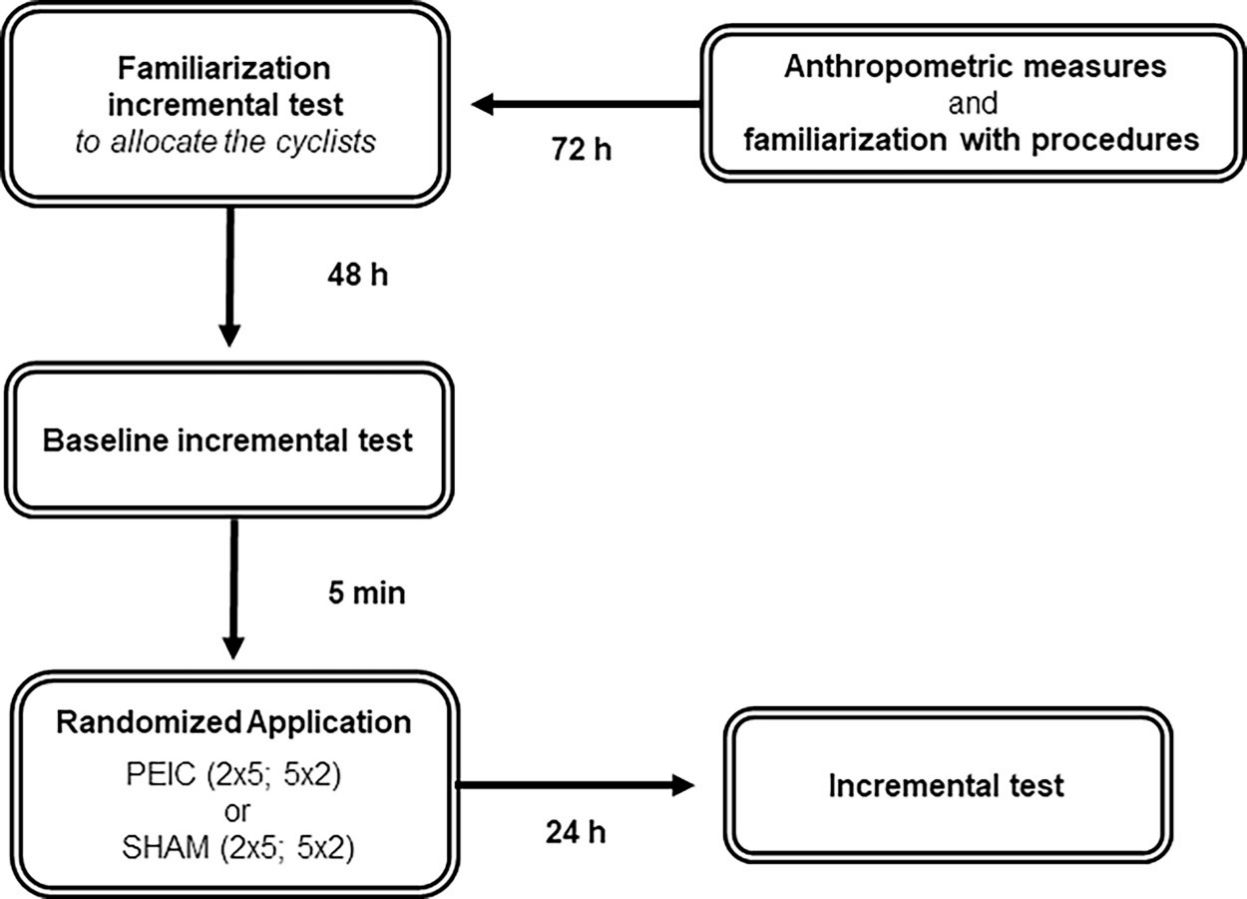
Experimental design of study. PEIC = Post-exercise ischemic conditioning; SHAM = cuff administration with low pressure.

This study was a randomized, single-blind (i.e., the tester was blind to the protocol interventions “received” by each cyclist) trial with PEIC and SHAM interventions. The cyclists were allocated into four different groups (PEIC 2 x 5; PEIC 5 x 2; SHAM 2 x 5; SHAM 5 x 2; n = 7 for each group) according to the counterbalanced peak power (W.kg^-1^) achieved in the familiarization incremental test [17]. All tests were performed in a constant environment (21.9 ± 1.3°C; relative humidity: 72.3 ± 7.9%). The cyclists attended the laboratory on four non-consecutive days, at the same time of day to prevent circadian influences [18]. At the first visit, anthropometric measures and all familiarization procedures (i.e., the cycle ergometer setup, cuff pressure measurements, and perceived scales training) were carried out. The cyclists were also asked to maintain their regular eating schedule, continue with this eating schedule throughout the experiment and were directed not to perform any moderate or intense physical exercise for 48 h before the first testing day. Coffee (or caffeine products), tea, and alcohol intake were prohibited, as well as strenuous exercise, for 48 h before testing. After 72 h, a familiarization incremental test to allocate the groups was performed. The baseline incremental test followed by PEIC or SHAM intervention was carried out 2 days afterwards, and the 24-h-post incremental test was performed on the next day (24 h post intervention). A blood sample (for creatine kinase analysis) was collected, and the lower limb muscle soreness and recovery level were also determined prior to each incremental test. After the baseline incremental test, to prevent a possible placebo and/or nocebo effect, which has been found in previous studies, we informed the cyclists that both interventions would improve their performance on the next test day and would cause no harm, despite the potential discomfort related to cuff administration [19–21]. Moreover, based on a prior study, the cyclists were asked about their “performance expectation” before the last incremental test: “Do you think your performance today will be similar, better, or worse in relation to your performance in the previous incremental test?” [22]. Fig 1 shows the experimental design of the study.

### Perceived scales and creatine kinase measurements

Before the incremental tests, the recovery level of the cyclists was evaluated through the subjective perception of recovery scale (PRS) [23]. The PRS is a validated and often-used tool for detecting athletes’ recovery level (i.e., a decrease in the PRS is related to impaired recovery) between training and competitions [23]. An experienced and trained professional performed the blood collection for CK analysis. Before the exercise, approximately 5 mL of venous blood was collected from the forearm. Blood samples were collected at baseline and 24 h post intervention [24,25]. The blood was carefully transferred to tubes and centrifuged at 3000 rpm for 5 min. After this step, the CK content was measured by commercially available analytical kits (Reagent Ck-NAC, Labtest Diagnostic S.A., Lagoa Santa, Brazil). Sample dosing was done using the LW200 Labtest. Furthermore, a visual analogue scale (VAS) was used to assess the cyclists’ muscle soreness at baseline and 24 h post intervention. On both days, the VAS assessment was performed before the blood collection and incremental testing. Through the palpation of the thigh and calf (anterior and posterior), the volunteers noted on the VAS the point that reflected their degree of soreness. The scale varied from 0 to 10 with the following markings: 0, no pain; 1–2, light pain; 3–7, moderate pain; and 8–10, severe pain [26]. The perception of pain in the PEIC and SHAM intervention groups was evaluated using the same scale. The analysis was performed immediately after the end of the intervention.

### Incremental test

All incremental tests were performed with the same cycle ergometer (Monark 839 E, Sweden). The height and saddle and handlebar positions were set up by the cyclists themselves and maintained during all sessions. The cyclists performed a 4-min warm-up at 40 W before the test, and the initial power was 40 W, which was increased by 20 W/min until voluntary exhaustion, keeping a constant cadence (80–90 rpm) [27,28]. The test was interrupted when the cyclist was unable to maintain a cadence of at least 80 rpm (for 10 s or more) or stopped voluntarily. The peak power output was estimated by multiplying the cadence by the total load (i.e., the 40 W at the beginning of the test plus 20 W/min for each stage).

### Heart rate, perceived exertion and power output

Throughout the incremental test, during the last 10 s of each stage, the heart rate (HR) (Polar, RS800CX, Finland), perceived exertion (RPE) (using a Borg scale [29] adapted to the cycling exercise as proposed by Robertson et al. [30]), and power output were recorded. When the subject was unable to continue the test, the HR recovery was recorded for 5 min in the dorsal decubitus position.

### Post-exercise ischemic conditioning intervention

All procedures lasted a total of 25 min, with a 5-min transition from the end of the incremental test and 20 min of the PEIC application. The PEIC intervention consisted of 2 cycles of 5 min of vascular occlusion with 5 min of vascular reperfusion or 5 cycles of 2 min of vascular occlusion with 2 min of vascular reperfusion. We used these settings based on the study of Beaven et al. [7] and to meet specific sports sciences settings [31,32]. Both procedures were unilaterally alternated and used a pressure of 50 mm Hg above the systolic blood pressure, which was previously measured by means of a sphygmomanometer (Aneroid G-TECH/Premium), of each subject [33] during the occlusion and 0 mm Hg during the reperfusion. The SHAM protocol was performed in the same manner as the PEIC protocol, but the occlusion was simulated with a pressure of 20 mm Hg. Blood flow was constantly checked by auscultation of the anterior tibial artery [21] during the interventions, and we confirmed the presence of proper occlusion/reperfusion phases. The occlusion was performed with a blood pressure cuff (77.0 cm length x 21.5 cm width) applied to the sub-inguinal region of the upper thigh and with the cyclists in a supine position.

### Statistical analysis

The normality of the data was tested with the Shapiro-Wilk test. The data are presented as the means ± standard deviation. To ensure similarity between the groups, the demographic and anthropometric characteristics, as well as the pain perception from the PEIC intervention, were analysed and compared through a one-way analysis of variance (ANOVA) test. A two-way ANOVA followed by the Bonferroni’s post hoc test was carried out to assess the interaction between time and the intervention. For the measurement of the correlations between the PRS and performance (time to exhaustion), Pearson's bivariate correlation test was performed. Cohen’s d effect size (ES) was calculated, with the magnitude classified as small (0.2), moderate (0.6) or large (1.2) [34]. A level of significance equal to 0.05 was adopted. The GraphPad (PRISM, 6.0, San Diego, CA, USA) program was used for the data analysis, and the G*Power Software (Dusseldorf, Germany) was used for the sample-size estimation.

## Results

All the cyclists completed the three days of testing. The data presented in Table 1 show the similarity between the groups (p > 0.05) before the interventions.

**Fig 2:**
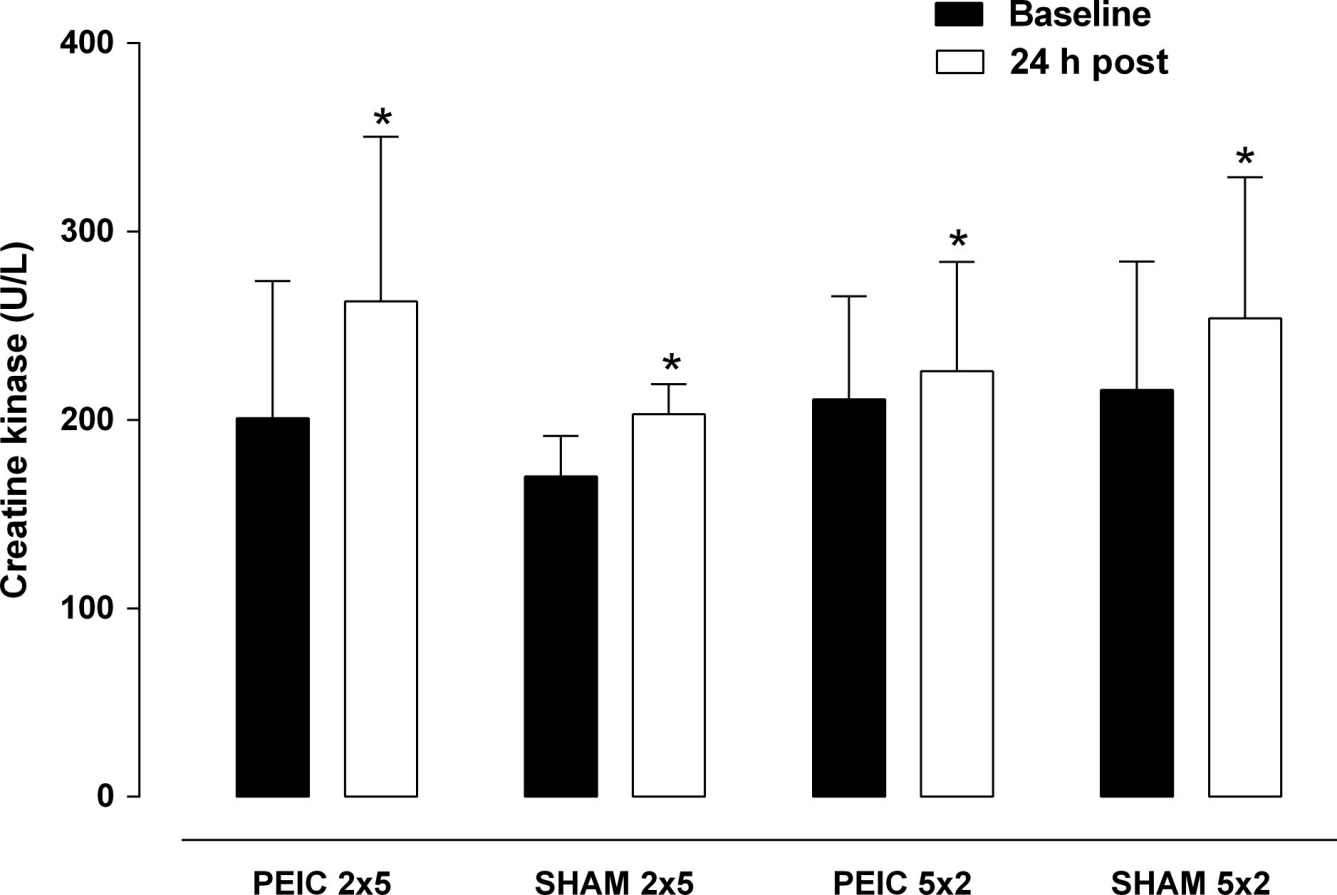
Creatine kinase at baseline and 24 h post intervention. PEIC = Post-exercise ischemic conditioning; SHAM = cuff administration with low pressure. * p < 0.05 = significantly different from baseline.

All the groups had increased levels of CK at 24 h post intervention compared with baseline (p < 0.05) (effect size: PEIC 2 x 5 = -0.32; SHAM 2 x 5 = -0.79; PEIC 5 x 2 = -0.11; and SHAM 5 x 2 = -0.20) (Fig 2). However, the mean CK was similar between the groups at both time points (p > 0.05) (baseline and 24 h post).

No differences in the perception of muscle soreness were found within the groups at baseline or at the 24-h-post time point or between groups (p > 0.05). The perception of recovery scores showed that the PEIC groups (2 x 5 and 5 x 2) felt more tired at 24 h post intervention (p < 0.05), but correlations were not found between changes in the PRS and changes in performance (time to exhaustion) (PEIC 2 x 5, p = 0.49; PEIC 5 x 2, p = 0.61; SHAM 2 x 5 p = 0.85; SHAM 5 x 2 p = 0.42). In addition, we pooled the data (n = 28) and did not find significant values (p = 0.37).

After 24 h of PEIC intervention, only the 5 x 2 SHAM group showed a reduction in the maximal HR. No other changes were observed in the mean, maximum and recovery HR values. Similarly, the power output did not differ at any time point. This information is displayed in Table 2.

**Table 2:** Results of the incremental test.

	SHAM 2 x 5		PEIC 2 x 5		SHAM 5 x 2		PEIC 5 x 2	
**Variables**	baseline	24 h Post	baseline	24 h Post	baseline	24 h Post	baseline	24 h Post
HR_Mean_ (bpm)	151 ± 7	151 ± 9	152 ± 11	150 ± 14	146 ± 11	145 ± 12	147 ± 11	150 ± 12
HR_Max_ (bpm)	197 ± 9	194 ± 5	196 ± 8	192 ± 11	191 ± 7	187 ± 7*	195 ± 8	197 ± 11
HR_Rec_ (bpm)	83 ± 5	85 ± 5	84 ± 6	83 ± 6	77 ± 4	77 ± 4	87 ± 6	86 ± 6
PO_Peak_ (W)	319.7 ± 43.4	306.1 ± 42.3	324.7 ± 34.3	323.3 ± 44.8	307.6 ± 44.6	303.7 ± 36.5	329.8 ± 48.6	334.6 ± 56.4
PO_Peak_ (W.kg^-1^)	4.1 ± 0.3	3.9 ± 0.4	4.1 ± 0.9	4.1 ± 1.0	4.2 ± 0.7	4.1 ± 0.6	4.3 ± 0.5	4.4 ± 0.6
RPE_Max_	9.9 ± 0.4	9.9 ± 0.4	9.6 ± 0.8	9.6 ± 0.8	9.6 ± 1.1	9.6 ± 1.1	9.7 ± 0.5	9.7 ± 0.5
PRS_mean_	7.0 ± 1.7	7.6 ± 1.7	8.3 ± 1.1	7.3 ± 1.1*	8.0 ± 3.0	7.3 ± 2.0	8.1 ± 3.0	7.1 ± 2.8*
MS_mean_	0.4 ± 0.8	0.7 ± 1.1	0.7 ± 1.0	0.4 ± 0.5	0.9 ± 1.2	0.8 ± 1.3	0.9 ± 1.5	1.0 ± 1.2

HR = heart rate; PO = power output; RPE = perceived exertion; PRS = perceived recovery; MS = muscle soreness. The data are the means ± SD. PEIC = Post-exercise ischemic conditioning; SHAM = cuff administration with low pressure.

*p < 0.05 = significantly different from baseline.

**Fig 3:**
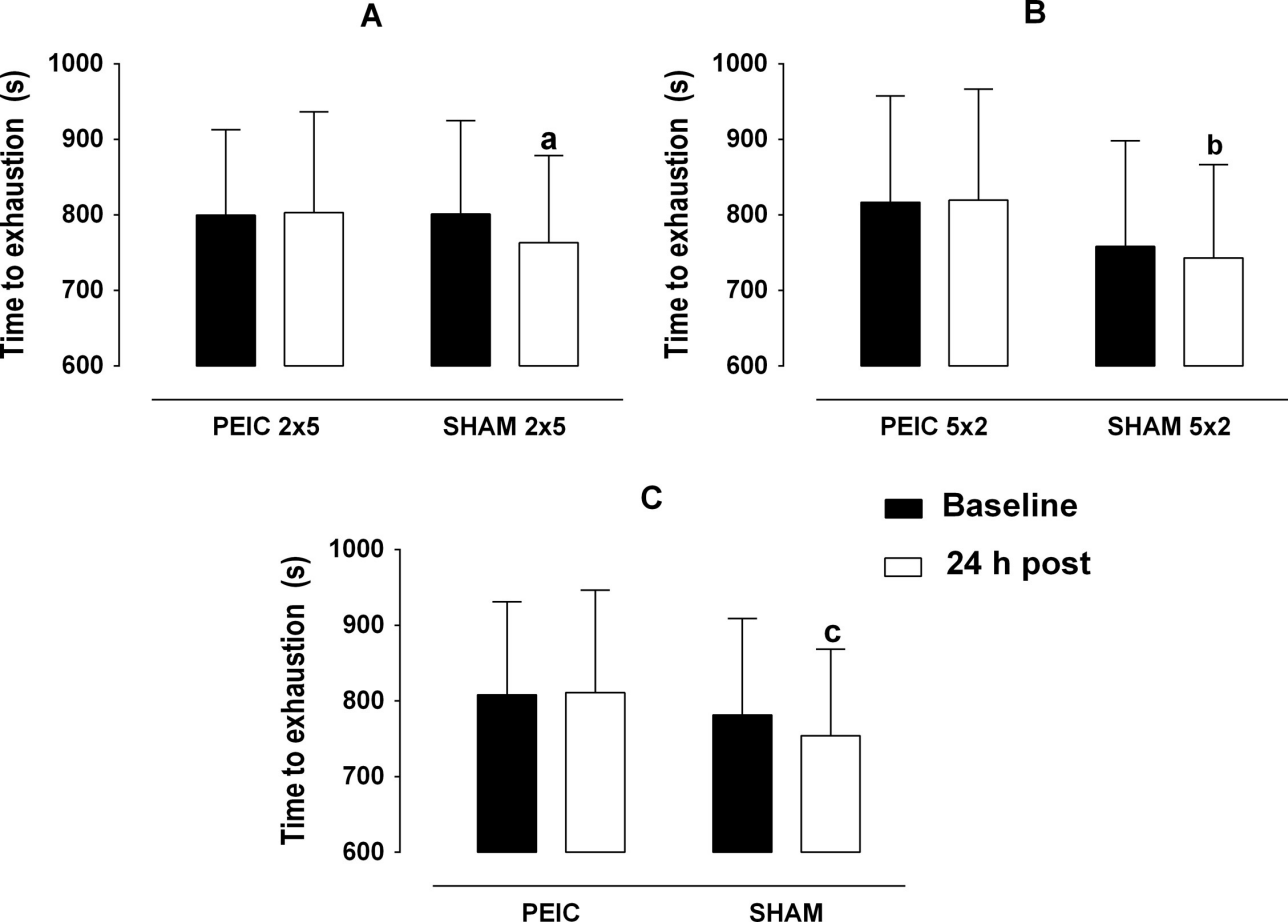
**Incremental test time to exhaustion at baseline and 24 h post intervention.** A: PEIC 2 x 5 (n = 7) and SHAM 2 x 5 (n = 7) groups; B: PEIC 5 x 2 (n = 7) and SHAM 5 x 2 (n = 7) groups; and C PEIC 2 x 5 and 5 x 2 (n = 14) and SHAM 2 x 5 and 5 x 2 (n = 14) groups. PEIC = Post-exercise ischemic conditioning; SHAM = cuff administration with low pressure; ES = Effect size. a: p = 0.015 from baseline ES = 0.32; b: p = 0.045 from baseline ES = 0.11; c: p = 0.02 from baseline ES = 0.27.

The PEIC groups had similar (p > 0.05) times to exhaustion in the incremental test 24 h post intervention (PEIC 2 x 5 = 3.3 s; PEIC 5 x 2 = 2.9 s) in comparison with baseline. On the other hand, both SHAM groups had decreased (p < 0.05) performance at 24 h post intervention [SHAM 2 x 5 = -37.9 s (4.7%); SHAM 5 x 2 = -17.0 s (2.2%)] (Fig 3A and 3B). Comparing the cyclists who underwent the PEIC (n = 14) and SHAM (n = 14) procedures, we found a decrement (p < 0.05) in the performance (24 h later) only in the SHAM intervention group (Fig 3C). No differences were found between the groups at baseline and 24 h post intervention (p > 0.05) (Fig 3A and 3B).

The perception of pain regarding the PEIC intervention was higher (p < 0.05) in the PEIC groups than in the SHAM groups (PEIC 2 x 5 (4.7 ± 2.1) = PEIC 5 x 2 (5.4 ± 2.2) > SHAM 5 x 2 (0.9 ± 1.2) = SHAM 2 x 5 (1.3 ± 1.6)). Table 3 shows the performance expectation and results of the incremental test.

**Table 3:** Percentage of performance expectancy about incremental test 24 h post intervention and the results of the test.

Cuff protocol		*Worse (%)*	*Similar (%)*	*Better (%)*
**PEIC 2x5**	*Expectation*	0	100	0
	*Result*	43	28.5	28.5
**PEIC 5x2**	*Expectation*	29	71	0
	*Result*	43	14	43
**SHAM 2x5**	*Expectation*	14.5	71	14.5
	*Result*	85.5	0	14.5
**SHAM 5x2**	*Expectation*	43	43	14
	*Result*	71.5	28.5	0

Values are in percentages of the total number of cyclists per group. PEIC: Post-exercise ischemic conditioning; SHAM: cuff administration with low pressure.

## Discussion

The purpose of this study was to evaluate the PEIC effect on exercise performance and physiological variables 24 h post intervention in trained cyclists. Our main finding was that the PEIC treatment prevented a decrement in performance 24 h after a maximal incremental cycling exercise test, possibly due a late effect of the ischemia-reperfusion intervention. This result supports our hypothesis and indicates that PEIC is a feasible strategy that can be applied to cyclists during a multi-stage exercise test, competition or training sessions.

The blood CK concentration is a biomarker of muscle damage that has been associated with a decline in muscle function and is used to identify muscle fatigue and schedule an appropriate recovery time [35,36]. In the current study, the CK increment was lower than that found in previous research [37], probably due to the low exercise volume (average 14 min) and the lower eccentric contraction from the incremental test performed here. Most important for the present context, PEIC did not influence the CK response 24 h after the incremental test was performed, which suggests that the PEIC-associated maintenance of performance 24 h after the incremental cycling test is not linked to the magnitude of muscle damage. Regarding this aspect, a recent study [9] has shown that PEIC intervention applied after a damaging bout of exercise led to decreased markers of muscle damage, such as CK, in recreationally active males. However, the CK responses in our study (Fig 2) do not support the possibility of “secondary” muscle damage.

An IR intervention could produce effects in two phases [14]: an early phase of protection, lasting between 3 and 4 h after reperfusion; and a late phase, beginning between 12 and 24 h after intervention. Plasma nitrate increases during the early phase [38], while iNOS expression (an isoform that synthesizes nitric oxide) increases during the late phase [39], and both can contribute to increased nitric oxide (NO) production; thus, we can expect increased mitochondrial efficiency [40] and a lower ATP cost for muscle production [41] due to the NO mechanism of action. Moreover, the NO could enhance exercise performance by diminishing the level of ROS [11], which is associated with reduced muscle fatigue [42]. Therefore, a possible increase in the NO concentration 24 h post IR intervention allows us to suggest a potential mechanism that could explain, at least in part, the beneficial delayed effects of PEIC on exercise performance.

Seeger et al. [43] applied an IR intervention before a 5-km time trial and reported that a runners group that had enhanced performance immediately after the IR intervention typically also had an improved performance 24 h after the IR. The mechanisms are still currently unknown. Although that IR intervention was before a running exercise, Page et al. [9] applied PEIC after a resistance exercise bout and observed an attenuated decrease in the maximal isometric voluntary contraction after 24 h. The author considered this result as an acceleration in recovery, but a possible 24-h effect on exercise performance cannot be refuted.

Interestingly, the PRS scores showed that both PEIC groups felt more tired at 24 h post intervention compared to baseline. Although the subjects felt more tired, their performance was similar to their baseline incremental test performance, in agreement with their performance expectations (Table 3). In contrast, the SHAM groups indicated a PRS condition 24 h post intervention that was similar to that at baseline but showed a decrement in performance, and no concordance with performance expectations was noted. Previous studies have reported significant correlations between the PRS and changes in performance [23,44], while we did not find correlations between changes in the PRS and changes in performance (time to exhaustion) in any group, either separately (for each group; n = 7) or when pooling the entire data (n = 28). Recovery is an interaction sensation that considers the psychological state before physical exercise in addition to the metabolic and physiological states [23]. Thus, since we evaluated amateur cyclists, we speculate that other factors, beyond specific test fatigue, could have influenced performance. Moreover, it is appropriate to note that the PRS scale utilized in this study, has been examined previously with other modes of exercise (e.g., high-intensity intermittent exercise) [23].

Performance may be influenced by intrinsic expectation [21]. In this way, the results of both PEIC groups agreed with their performance expectations regarding the performance test, but the results of both SHAM groups did not (Table 3). At first glance, these results may indicate that the SHAM interventions did not produce a placebo effect, but a clear explanation could not be provided due to the lack of consensus about SHAM groups.

No previous study has evaluated the effect of different PEIC protocols. Some studies showed that the time and number of IR cycles may influence the preconditioning effect [6,45]. A long IR protocol (i.e., 8 cycles x 5-min) before exercise did not show an additional preconditioning effect compared to smaller cycles (i.e., 4 cycles x 5-min), and this regimen was no different from a sham intervention [6]. However, our results demonstrated that protocols with different time durations (PEIC 2 x 5 and 5 x 2) were efficient at preventing decrements in the performance of the cyclists, which is in contrast to the results of a previous study [6] in which an IR protocol involving a long time was followed by performance decrements.

Only three studies have investigated the effect of PEIC on performance 24 h post intervention [7–9]. While Beaven et al. [7] analysed the effect of PEIC immediately and at 24 h after a strenuous exercise and found a beneficial effect on power and sprint performance immediately and 24 h after the cycles of PEIC, Page et al. [9] described an attenuation of the decrease in the maximal isometric voluntary contraction 24, 48 and 72 h after applying PEIC post eccentric exercise. Although these findings corroborate our data, Northey et al. [8] did not find improvements in performance 24 h later on squat and countermovement jump height after PEIC. These different outcomes might be due to an influence of some variables, such as the level of training (well trained [8] or recreationally active [7,9]). It is hypothesized that well trained subjects could have different responses to an IR intervention compared to less trained subjects [46].

In this study, we did not measure total serum antioxidants to assess ROS and biomarkers, which are related to changes in work economy (e.g., blood lactate) or muscle strength and power. Such measurements could be the focus of future investigations.

In conclusion, both acute PEIC interventions promoted the maintenance of exercise performance 24 h after a cycling test until exhaustion. Our findings support the use of PEIC as a feasible approach that can be applied during intervals or training sessions or in competitions occurring on consecutive days.
